# Progress in providing legal abortion services after law reform: A quantitative study in three provinces of Argentina

**DOI:** 10.1371/journal.pgph.0003526

**Published:** 2025-02-25

**Authors:** Sarah C. Keogh, Georgina Binstock, Mailén Pérez Tort, Susheela Singh

**Affiliations:** 1 Guttmacher Institute, New York, New York, United States of America; 2 Centro de Estudios de Población, Buenos Aires, Argentina; 3 Consejo Nacional de Investigaciones Científicas y Técnicas (CONICET), Buenos Aires, Argentina; California State University San Marcos, UNITED STATES OF AMERICA

## Abstract

Argentina’s 2021 abortion law grants the right to abortion on-request up to 14 weeks’ gestation, as well as continuing to allow abortion after 14 weeks on specific grounds. The early years after law reform provide a unique opportunity to assess progress and identify barriers, to both inform program improvements and guide other countries undergoing reform. This study assesses the first two years of law implementation. We surveyed a purposive sample of 45 key informants about implementation successes and barriers. In addition, we surveyed 223 public health facilities (selected through stratified systematic random sampling) in three provinces: Buenos Aires, Chaco and La Rioja. We collected information on abortion services, resources, personnel, training, and obstacles to provision. We present weighted results on characteristics of abortion provision by facilities, representative of each province, complemented by key informant perspectives. Two years into law reform, abortions under 14 weeks were offered in a large number of facilities at all levels, while later abortions were offered mainly in hospitals. Facilities adhered to protocols, had adequate supplies, and kept comprehensive records. Over 90% of abortions were performed using misoprostol, with MVA accounting for most of the remainder. Major barriers to provision included insufficient personnel, exacerbated by high levels of conscientious objection (over 60% of hospitals had at least 2 objecting doctors), and inadequate training in methods other than misoprostol, particularly among health centers. Argentina has made impressive advances in the short time since law reform. Implementation could be improved by increasing personnel (through incentives, task-shifting, and enforcement of conscientious objection regulations), strengthening training on different abortion techniques, and expanding public information campaigns about abortion rights and services available. In the face of diminished support for abortion under the new government, measures to strengthen abortion services and reduce stigma are critical, if reproductive rights are to be upheld.

## Introduction

In December 2020, Argentina’s Congress passed the Law 27.610 on Voluntary Interruption of Pregnancy (Interrupción Voluntaria del Embarazo or IVE hereafter) giving the right to women and people of other gender identities with gestational capacity to freely access abortion up to 14 weeks’ gestation without giving a reason, at the primary and secondary care levels. This landmark decision made Argentina the third, and most populous, country in Latin America to legalize abortion on request in the first trimester of pregnancy. Additionally, the law provides for Legal Interruption of Pregnancy beyond 14 weeks (Interrupción Legal del Embarazo or ILE hereafter) under specific circumstances such as rape or health risks [1].

Prior to the new law, early abortion was available under the restricted circumstances of rape or health risks, but was very difficult to access in practice, with anti-abortion groups often interfering with its implementation. This led women to resort to clandestine (often unsafe) abortions, with their associated health and social consequences [2–7]. While estimates of abortion incidence are scarce and based on indirect measures [8], official statistics indicate that 53,000 women were hospitalized due to abortion in 2013, 15% of them adolescents [9]. Despite the passing in 2002 of the Sexual Health and Responsible Procreation Law guaranteeing access to information, orientation, contraception, and reproductive health services, abortion continued to be a hidden practice with professionals and pregnant women risking criminal prosecution, even if they complied with all the applicable legal requirements [10]. Social and feminist activists became crucial in publicizing cases of people who had been denied access to legal abortions [11], leading to a number of court cases and ultimately a Supreme Court judgment known as FAL/12, that set the ground for the decriminalization of abortion in all rape cases without a prior judicial procedure, and stating that health risks must be understood in a broader sense. As Fernández Vásquez and Brown (2019:67) state, “Between 2007 and 2017, the legal context of abortion in Argentina changed from a restrictive to a permissive interpretation which probably contributed to greater social acceptance of decriminalization” [12]. Thus, feminists, social, and human rights organizations played a pivotal role in changing the social climate toward abortion and initiating a massive movement demanding legal, safe and free abortion that eventually resulted in the 2020 legal reform.

The new abortion law heralds significant progress in women’s health, reproductive rights and autonomy. It guarantees the right to receive free care, access the procedure within 10 days, respectful treatment, privacy and confidentiality, accurate and accessible information, as well as free provision and fitting of contraceptive methods (under law 25.673). The Ministry of Health protocol for abortion care outlines techniques for performing abortions following safety and quality standards, including medication abortion (misoprostol alone or with mifepristone), and surgical procedures (manual vacuum aspiration (MVA) and dilation and evacuation (D&E)) [1]. All of these methods are recommended by the World Health Organization, with D&E specifically indicated for abortions at or above 14 weeks’ gestation [13]. In Argentina, misoprostol has been the main method favored by the government for abortions under 14 weeks since the enactment of Law 27.610 [14,15]. At the time of the study, mifepristone had not yet been approved in Argentina (it was approved in March 2023); however, some facilities were able to provide it before official approval thanks to a waiver [16].

Government provision of abortion care through the public sector is critical for increasing abortion access, particularly for those who cannot afford private sector services: one third of the total population and two thirds of the more disadvantaged groups rely solely on the public system for healthcare (Authors’ calculations based on the Permanent Household Survey, 4^th^ trimester 2022 [17]). Despite additional challenges brought on by the Covid-19 pandemic for a public health sector with already limited capacity and resources [18], government agencies worked to ensure effective and widespread implementation of the law, applying service delivery protocols, training providers for quality care, coordinating the operational aspects of integrating abortion services into public facilities, and educating the public about the rights and responsibilities established by the law. Public-sector abortion provision in Argentina has increased from 73,487 abortions in 2021 to 96,664 in 2022 [14,15].

The early years after law reform provide a unique opportunity to assess progress, identify implementation barriers and work towards solutions. These assessments can inform efforts in Argentina to ensure that access to quality safe abortion is available for all in need and provide guidance to other countries implementing abortion law reform. Studies monitoring the implementation of the 2018 law liberalizing abortion in Ireland have been critical in informing further policy-making and programming to improve access [19–21]. Efforts to monitor the implementation of new abortion regulations are similarly being undertaken in Uruguay [22] and Colombia [23]. In Argentina, the *proyecto mirar* (“Looking forward”) is an initiative to monitor implementation of the abortion law across four dimensions (enabling environment, supply, demand, and quality), based primarily on official data [24]. This initiative, however, does not include facility-based data on resources, personnel, training, and other aspects that are crucial to document in order to identify advances and obstacles in the provision of quality abortion services. The present study contributes to filling this evidence gap on implementation of the Argentina law reform, through health facility surveys in three provinces and key informant interviews; these new data provide an in-depth assessment of successes and challenges encountered in the first two years of implementation, separate from official data. Policymakers and healthcare professionals within Argentina can find a comprehensive discussion of survey findings and policy recommendations in a Spanish-language report [25]. This article provides a deeper analysis of barriers related to staffing, supplies and information dissemination, drawing out their relevance and implications for law reform efforts globally, and contextualizes the findings within the broader literature on abortion law reform in Argentina, Latin America and other regions.

The study was conducted in the provinces of Buenos Aires, Chaco, and La Rioja, which represent diverse socioeconomic profiles, sexual and reproductive health indicators, sociocultural norms, and levels of preparedness to provide abortion. Our aim in including these three provinces was to examine how implementation progress and challenges may differ according to factors such as remoteness, level of health system development, and sociocultural characteristics, especially given the federal system and concomitant decentralization of government processes in Argentina. Buenos Aires is the most populous and economically developed province, housing 38% of the country’s population, and with a total fertility rate of 1.9 in 2019 [26]. Despite being the economic engine of Argentina, it faces challenges in terms of inequality and poverty: Greater Buenos Aires (GBA) has nearly half of its population below the poverty line and has sparse healthcare coverage (0.8 facilities per 10,000 inhabitants). The rest of the province of Buenos Aires (RPBA) fares slightly better with 30-40% below the poverty line and 1.6 facilities per 10,000 inhabitants [17], but has a less open social climate regarding abortion than GBA. In 2022, 40,880 abortions were registered in the province’s public health system, up from 5,028 in 2020 prior to law reform [15]. However, this does not account for abortions that occurred outside the formal health system.

La Rioja is similar to Buenos Aires in terms of poverty (36% below the poverty line) and fertility (1.8 in 2018), but has better healthcare coverage (4.4 facilities per 10,000 inhabitants) [27]. However, the density of providers per population does not account for difficulty of access: in remote sparsely populated regions, it may be more difficult to access facilities despite having a relatively higher number of facilities per inhabitant. A strongly Catholic province, La Rioja has a very conservative social climate. Abortion is highly stigmatized, and until 2020 only three mobile health teams provided abortions, while 90% of health professionals declared themselves conscientious objectors [28]. In the first two years of law reform, 1,773 abortions were registered [15], up from only 50 in 2020 [29]. Chaco is one of the poorest provinces, with 60% of the population in its capital living below the poverty line and 1.2 healthcare facilities per 10,000 inhabitants [27]. Its total fertility rate (2.4 in 2018) is one of the highest in the country (Estimations based on births and population projections from the Dirección de Estadísticas e Información de la Salud (DEIS)). In the first two years of law reform, 1,963 abortions were registered in the public system [15]. Of the 11 court cases against the new law registered in provincial settings, two were filed in Chaco, indicating strong resistance in the province [15,30].

While these diverse jurisdictions are not nationally representative, they provide a useful snapshot of how law implementation and barriers encountered may differ across the country.

## Materials and methods

### Data collection

We conducted a survey of key informants (KIS) and a survey of public health facilities (HFS), to obtain different perspectives and information on implementation of various aspects of abortion services. While both surveys are quantitative, the aspects covered are different and complementary, with the facility survey covering service provision on the ground, and the key informant survey covering implementation successes and challenges at the national and local government levels. Combining the two data sources thus provides a multifaceted picture of the state of abortion implementation at all levels of the health system. Fieldwork was conducted from July 2022 to January 2023.

#### Key informant survey.

The objective of the KIS was to document knowledgeable professionals’ perspectives on successes and barriers in implementing the new law. The KIS was administered to a purposive sample of 45 professionals with knowledge and experience on abortion at the national and provincial level. The sample was built using the snowball method. Initially, individuals were chosen based on their roles in healthcare, government, research, or civil society groups. They were then asked to provide references to others with relevant experience. The final sample included individuals from the healthcare system, Ministry of Health, academia, and civil society organizations, all of whom are relevant actors in providing information across various dimensions of abortion implementation at the national or provincial level. The sample comprised 16 healthcare professionals involved in management and direct interaction with patients, 14 government officials, 8 members of civil society organizations, 2 academics, and 6 individuals engaged in diverse activities. The questionnaire addressed abortion service availability, compliance with official guidelines, staff training, barriers to access, opinions on the role of health authorities at national and provincial levels, and recommendations to improve service quality and accessibility. Interviews were conducted virtually (using Zoom) or in person in a private room, and lasted approximately 1 hour. They were recorded subject to the respondent’s written informed consent.

#### Health facility survey.

The Health Facility Survey (HFS) gathered information from administrators or doctors in charge of abortion provision, to understand progress and obstacles in delivering quality services in compliance with the law. The HFS was administered in 223 public-sector health facilities in three provinces: Buenos Aires, Chaco, and La Rioja. Sampling was stratified by province and facility type, selecting 30% of hospitals and 25% of health centers from our sampling frame for each province through systematic random sampling. Since the main focus of the study was to assess implementation and quality of care within abortion-providing facilities, rather than determine coverage of abortion services across all facilities within provinces, our goal was to draw our sample from the universe of abortion-providing facilities. We were able to access the Ministry of Health’s list of 472 registered providing facilities in the province of Buenos Aires, which was particularly important given there are around 2000 facilities overall in this province. However, we were not able to get a list of providing facilities for Chaco and La Rioja, so the sampling frame for these two provinces is the list of all facilities (with abortion provision status unknown). As there are fewer facilities overall in these provinces, it was less problematic to sample from the full universe of facilities (Table 1). Given the large size and diversity of the Province of Buenos Aires, we divided the sample into two jurisdictions: Greater Buenos Aires (GBA) excluding the City of Buenos Aires (a separate jurisdiction of less interest to this study because it has the most resources for implementation and most advanced monitoring), and the rest of the province (RPBA).

**Table 1:** Sampling frame and sample for the health facilities survey.

	Hospitals		Health Centers		Total	
Jurisdiction	Sampling Frame	Sample N	Sampling Frame	Sample N	Sampling Frame	Sample N
GBA	68	23	184	44	252	67
Rest of PBA	78	29	142	35	220	64
Chaco	52	21	96	24	148	45
La Rioja	33	19	54	28	87	47
**Total**	**231**	**92**	**476**	**131**	**707**	**223**

The HFS questionnaire covered basic facility characteristics, abortion provision, number and characteristics of procedures, staff training, provider attitudes and beliefs, obstacles during the COVID-19 pandemic, and perceptions of women’s knowledge about the law and abortion methods. Interviews were conducted in person by trained researchers in a private room at the facility and lasted approximately 45 minutes.

### Ethics statement

Before all KIS and HFS interviews, respondents signed an informed consent form explaining the study and their right to end the interview at any time. The study was approved by the Research Ethics Committee of the Argentine Ministry of Health’s Directorate of Health Research (*Dirección de Investigación en Salud*) by letter dated May 17^th^ 2022, as well as the Guttmacher Institute’s Institutional Review Board (IRB00002197).

### Data analysis

Data from the KIS, including open responses, were entered and analyzed in Excel. Data from the HFS were analyzed in STATA and SPSS using descriptive statistics and cross-tabulations to compute the distributions and characteristics of abortion services by province and facility type. In the analysis, we present estimates separately for municipal (secondary-level) hospitals and province-level (tertiary) hospitals, as they tend to have distinct roles and characteristics regarding abortion provision. Missing observations were minimal (<1%) and missing at random; they are excluded from estimates.

As the study was not nationally representative, we do not present overall estimates of all 4 jurisdictions combined. Instead, we present estimates by province (weighted to be representative at the province level) and by facility type, to provide a snapshot of abortion service provision in diverse geographical/cultural contexts and facility levels. Because our facility sample in Chaco province included only 5 facilities that provided abortion, analyses of the subsample of abortion-providing facilities do not include separate estimates for Chaco given its small number of cases. Estimates among abortion-providing facilities are therefore presented for 3 jurisdictions: GBA, RPBA, and La Rioja. As our aim was not to evaluate the significance of differences between provinces or facility types, we do not present any significance tests.

An advisory panel of ten members from academic, healthcare, government, and civil society communities provided input on the work plan, survey questionnaires, and results.

## Results

### Abortion provision landscape

In the government list of facilities registered as providing abortion in Buenos Aires province used to draw the sample, just over half (55% in GBA, 52% in RPBA) provided only IVE (up to 14 weeks), while 33% and 36% provided both IVE and ILE (beyond 14 weeks); the remainder (12%) did not provide abortions despite being on the list of abortion-providing facilities. If proportions providing abortions were expressed as a percentage of all facilities in GBA and RPBA (including those not registered as providers), assuming the register is accurate, the corresponding proportions would be 15% providing only IVE and 9% providing both IVE and ILE in GBA, and 11% providing only IVE and 7% providing both IVE and ILE in RPBA.

As explained in the methods section, the facility sampling frame for Chaco and La Rioja included all facilities regardless of their official abortion provision status. In La Rioja, 30% provided only IVE, and 10% provided both IVE and ILE. In Chaco, proportions were much smaller: 4% provided IVE alone, and 5% provided both types.

**Fig 1:**
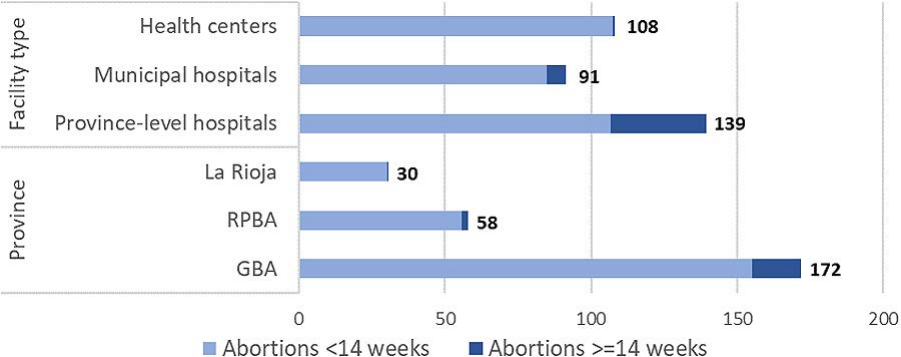
Average number of abortions per facility in last six months among abortion-providing facilities.

Reflecting official guidelines, ILE was mainly provided by hospitals (around half of surveyed hospitals offered ILE, against 14% of health centers). Among health centers, 48% provided IVE only, while 38% did not provide any abortions. The lower proportion of health facilities providing abortion is explained by the establishment of referral systems to centralize abortion provision across districts. Key informants described how health centers that do not provide abortions refer to (generally) province-level hospitals. Consequently, these hospitals perform the highest volume of abortions (on average 139 procedures over the past six months) (Fig 1). While this means that some women will have to travel further to access abortion, the majority of providers reported that most service users live within 10 km of the facility. However, it is difficult to know how many would-be users did *not* access a facility because of distance.

In all three provinces, more than 90% of the abortions performed were less than 14 weeks gestation. Almost all abortions were under 14 weeks in health centers (99%) and municipal hospitals (94%), while in province-level hospitals a higher proportion of abortions (24%) were at gestations of 14 weeks or more (Fig 1).

### Implementation successes

Despite the additional challenges created by the COVID-19 pandemic in terms of service access restrictions, disrupted supply chains and decreased personnel, the public sector offered services free of charge with minimal method stock-out problems. Several successes in these first two years of implementation are worth highlighting.

#### Adherence to protocols.

Between 60–70% of KIS respondents agreed that both hospitals and health centers guarantee confidentiality and privacy during care, carry out the practice within 10 days of being requested and do not require a police report in case of rape or presentation of the Argentinian National Identity document. Informants positively evaluated the Ministry of Health’s role in the provision of misoprostol, quality training, informational materials, and the 0800 “Sexual Health” hotline (which provides personalized guidance and support to the general population and healthcare teams, to facilitate access to services nationwide). However, respondents indicated that informational materials are mostly digital, with very little paper materials to distribute, and saw this as a gap that should be filled.

#### Comprehensive data records.

Results from both surveys highlighted the need for timely reporting of facility-level abortion statistics, in order to restock medication such as misoprostol. Over 90% of facilities used the medical history form, and under 20% (also) used the Perinatal Computer System (SIP). Most facilities collected basic data on abortion method (90-100%), weeks’ gestation (79-93%) and patients’ age (79-91%). While the Voluntary and Legal Termination of Pregnancy Information System (SILVE, introduced by the government in 2021) will enable more efficient tracking and comparisons of abortion characteristics across the country when fully operational, at the time of the survey it was still in a pilot phase, and was used by less than 5% of facilities (mostly hospitals), and only in GBA and RPBA [14,15].

#### Stock of methods and equipment.

Nearly all abortion-providing facilities offered misoprostol for abortions up to 14 weeks (Table 2). Misoprostol for later abortions was mainly offered by province-level hospitals (74%), with only 8% of health centers offering it. In the six months preceding the survey, misoprostol was used for 98% of abortions in health centers, 90% in municipal hospitals, and 75% in province-level hospitals (which tend to receive later abortions more likely to require a surgical procedure). Mifepristone was only officially registered for commercial use in Argentina in March 2023, so at the time of the survey the combination of mifepristone and misoprostol was still not widely offered. Most health facilities obtained misoprostol supplies from the provincial government, and respondents reported that the quantity supplied was generally sufficient.

**Table 2:** Methods offered by gestation, availability of trained personnel, and proportion of abortions performed with each method, by province and facility type, among facilities that provide abortion services.

		Province			Facility type		
		GBA	RPBA	La Rioja	Province-level hospitals	Municipal hospitals	Health centers
**Misoprostol**	% facilities that offer for <=14 wks	96.2	100.0	100.0	97.4	100.0	97.2
	% facilities that offer for >14 wks	30.1	24.7	30.9	74.0	45.1	8.3
	% facilities that have trained staff	100	100	100	100	100	100
	% abortions performed with method	91.8	94.3	96.1	77.1	90.3	99.1
**Misoprostol &** **mife** pristone*	% facilities that offer for <=14 wks	47.9	40.4	57.5	81.5	47.6	35.3
	% facilities that offer for >14 wks	23.5	19.1	21.0	65.5	34.9	4.1
	% facilities that have trained staff	74.0	86.2	87.5	93.5	94.0	73.8
	% abortions performed with method	2.3	1.1	3.1	6.9	2.2	0.3
**MVA**	% facilities that offer for <=14 wks	28.2	33.9	20.5	63.2	76.6	7.6
	% facilities that offer for >14 wks	23.7	19.8	15.5	61.4	41.3	3.5
	% facilities that have trained staff	59.1	64.4	60.6	84.8	95.6	45.6
	% abortions performed with method	4.6	3.8	0.8	12.3	6.9	0.4
**D&C**	% facilities that offer for <=14 wks	14.0	20.5	10.0	34.4	50.7	2.8
	% facilities that offer for >14 wks	13.8	12.7	5.0	32.0	31.1	1.4
	% facilities that have trained staff	63.2	50.3	50.0	82.4	89.6	40.8
	% abortions performed with method	1.0	0.5	0.0	2.9	0.6	0.0
**D&E**	% facilities that offer for <=14 wks	15.4	12.7	5.0	35.9	31.5	1.4
	% facilities that offer for >14 wks	19.7	11.3	0.0	42.2	23.4	2.8
	% facilities that have trained staff	59.8	48.0	39.3	75.6	84.6	38.5
	% abortions performed with method	0.3	0.3	0.0	0.8	0.0	0.1

*The combined method (mifepristone and misoprostol) was approved in March 2023, after fieldwork was completed; however, some facilities were already providing this method before official approval thanks to a waiver.[16].

While one or more surgical methods were available in up to 77% of hospitals, and an even higher proportion of facilities had personnel trained in these methods, in practice only a small proportion of abortions were performed with surgical methods, mostly MVA (Table 2), and very few with D&E and the non-recommended D&C (Table 2). While most hospitals were satisfied with their supply of MVA kits, satisfaction dropped to 64% among health centers. Moreover, 99% of all health centers received no kits; they are generally not expected to perform MVAs.

All abortion-providing facilities reported offering postabortion contraceptive counseling, and over 90% provided pills, injectables, implants, condoms, and intrauterine devices free of charge. Tubal ligation was offered free of charge in 60-77% of hospitals, but only 16% of health centers. While the majority of facilities reported that “most patients” adopted a contraceptive method post-abortion, around a quarter of municipal hospitals and health centers reported that “over half” of users did not. Some key informants suggested incorporating counseling on long-acting contraception from the beginning of the abortion process, rather than waiting till the post-abortion follow-up appointment (which many health center patients reportedly did not return for).

### Remaining implementation challenges and potential solutions

#### Abortion provider capacity.

Official guidelines require a doctor to provide or sign off on all abortions (whether MA or surgical). On average, each abortion providing facility had 2.6 physicians, with the largest number in province-level hospitals (5.6), followed by municipal hospitals (2.9) and health centers (1.7). While the majority of facilities reported having an adequate number of physicians to meet demand, 15% of municipal hospitals and 32% of province-level hospitals and health centers reported insufficient numbers, particularly in GBA (38% of facilities).

According to KIS respondents, the greatest obstacle to effective implementation is the lack of trained personnel, including physicians, gynecologists and anesthetists. A third of key informants suggested increasing the number of professionals, by for example implementing incentive policies or improving working conditions to attract more candidates. Some proposed expanding legal provision to mid-level staff, and mentioned that there is currently a movement to allow midwives to provide abortions.

#### Abortion provider training.

During 2022, 83% of province-level hospitals, 70% of municipal hospitals and 68% of health centers providing abortion reported that their staff received training on abortion-related topics. While the training was considered sufficient by the majority of facilities, several gaps were identified. First, HFS respondents highlighted an urgent need to extend training to health personnel beyond medical staff (mentioned by 23-32% of facilities). More than half of KIS respondents suggested awareness and values clarification training for all personnel, starting with staff at the front desk. The second most cited training gap in health centers was the use of mifepristone combined with misoprostol (25%). Although all facilities had one or more physicians trained in the use of misoprostol, a substantial proportion did not have any professionals trained in the mifepristone-misoprostol combination, particularly in health centers (26%). In hospitals, the second most cited training gap was second-trimester abortion techniques (19-23%). MVA training was requested by 13% of municipal hospitals, and 19% of health centers.

#### Conscientious objection.

According to KIS respondents, the second biggest obstacle to implementation (mentioned by 17%) related to attitudinal, ideological and religious opposition. Another 10% mentioned professionals’ imperfect knowledge of the legislation, leading to lack of compliance as well as fear of stigma, social condemnation, or legal action.

**Fig 2:**
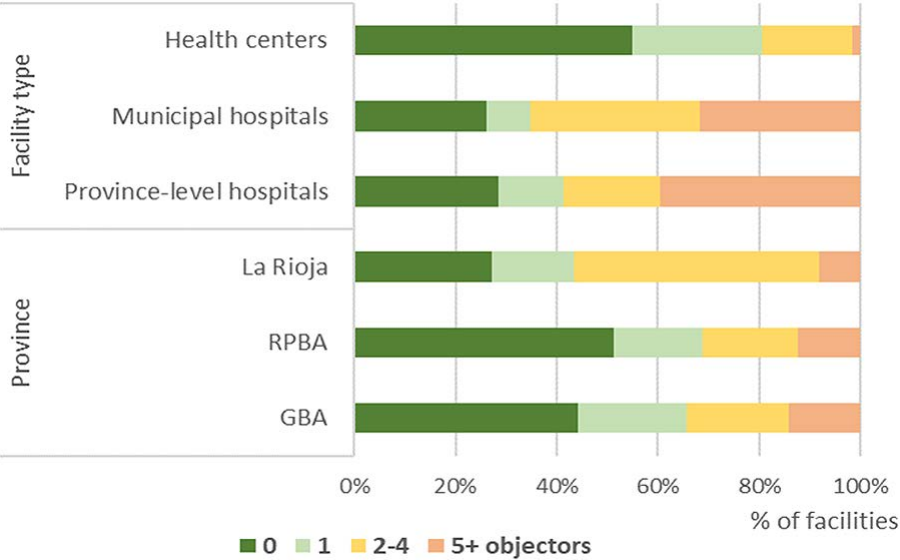
Percentage of facilities reporting conscientious objection among physicians, according to number of objecting physicians, by province and facility type.

The right to conscientious objection (CO) established in article 10 of Law 27,610 can only be exercised by individual professionals who directly participate in the termination process, and should not imply the denial or obstruction of the practice, which should be guaranteed according to the referral protocol. Among abortion providing facilities, conscientious objectors were found among professionals involved in abortion care in 71-74% of hospitals and 45% of health centers, particularly in La Rioja (Fig 2). Nearly half (47%) of province-level hospitals and 33% of municipal hospitals registered five or more objecting physicians. In addition, 55-65% of hospitals and 18% of health centers reported staff claiming CO other than physicians – mainly nurses, but also administrators, psychologists, social workers, health agents, anesthetists, laboratory technicians, surgical technicians and sonographers. KIS respondents confirmed that it is common for non-medical staff to declare themselves conscientious objectors despite not having the legal right to do so. This can present a significant barrier to accessing abortion in facilities with otherwise willing physicians.

To minimize the effect of CO on service provision, providers chose to form interdisciplinary teams with those who did not object, thus guaranteeing abortions. Guidelines for performing safe abortions emphasize the importance of having interdisciplinary teams spanning medicine, social work and psychology to provide comprehensive, people-centered care [31], and 77-83% of facilities had an interdisciplinary team in place, despite the high prevalence of CO.

Some key informants indicated a need for greater regulation of non-compliance (in the case of illegal CO). To increase employees’ understanding of the limits of the law, they suggested implementing mandatory training on CO for all staff. Several informants suggested considering CO during hiring; a few suggested a registry of conscientious objectors, but others came out strongly against this, fearing it would restrict providers’ freedom to reconsider their position. Informants suggested it might be useful for professionals to be able to specify in which situations CO is invoked, since many would not object to terminations of less than 10 weeks.

#### Facilities that do not provide abortion services.

A third of abortion-providing facilities indicated that they had sometimes been unable to provide abortions, with important differences between areas (50% in GBA, 17% in the RPBA, and 11% in La Rioja) and by facility type (35% in province-level hospitals and health centers, and 19% in municipal hospitals). Inability to provide the service was mostly in the case of second-trimester pregnancies that the facility did not have the trained personnel to attend to, and only occasionally due to lack of space, instruments, or doctors’ temporary absence.

**Fig 3:**
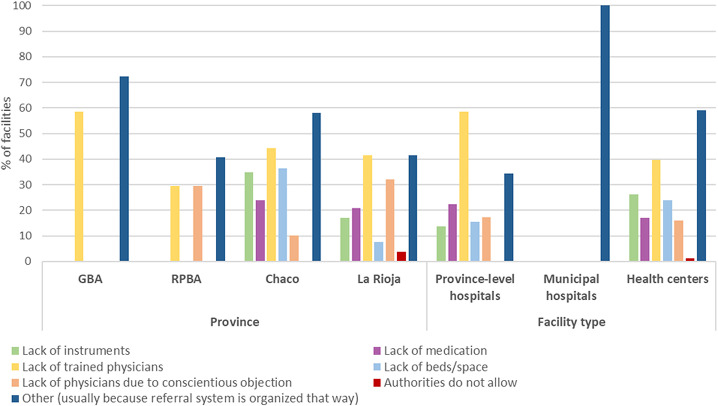
Percentage of non-providing facilities according to specific reasons for not providing abortion services.

Among surveyed facilities that did *not* provide abortion *at all*, the most frequent reason, particularly in health centers, was that the facility is organized to refer to a district or province-level provider in order to centralize abortion provision (Fig 3). The referral system is implemented across all three provinces when a woman seeks the procedure at a facility that does not provide it. However, the approach varies significantly between facilities depending on the type of services they provide. It may range from simply providing information about the nearest provider to offering counseling, scheduling an appointment, and coordinating arrangements. These variations largely depend on the staff at each facility, which can either facilitate or hinder access to services. Consequently, many respondents emphasized the critical importance of ensuring that abortion-related training is provided to all facility personnel. Such training would ensure that staff are well-informed and sensitized, enabling them to effectively assist or refer patients seeking the procedure.

All non-providing facilities indicated that they referred users to another facility: mainly to hospitals in Chaco and La Rioja, and to hospitals or health centers in GBA and RPBA. Facilities had referred on average between 5 (La Rioja) and 18 (RPBA) individuals requesting abortion services in the last six months. However, KIS respondents mentioned problems with the referral system, including the lack of adaptation of services to demand and the arbitrary gestational limits for abortions over 14 weeks, as barriers to implementation.

The lack of trained physicians was the second most important reason for not providing at all (30-60% depending on province), and the main reason in province-level hospitals (58%). The lack of physicians due to conscientious objection was cited as a reason for not providing abortions by 30-32% of facilities in La Rioja and RPBA. Among non-providing facilities, only around a third of province-level hospitals and health centers (and no municipal hospitals) planned to start offering abortion in the future.

#### Obstacles faced by abortion patients.

When asked about the main obstacle that patients encounter in accessing abortion services, KIS respondents predominantly mentioned insufficient numbers of facilities (21%), patients’ lack of knowledge (19%), lack of information educating people about their rights (14%) and social stigma (9% - especially in la Rioja). This is supported by health providers’ reports that a significant proportion of patients do not know any abortion method (though most have heard of misoprostol), and many do not know they can request a termination under 14 weeks without giving a reason. Some 78% of KIS respondents negatively evaluated the Ministry of Health’s national dissemination campaigns, and suggested the need for more sustained public information campaigns, so that people know their rights and where and how to access services.

## Discussion

While a large proportion of countries worldwide still have restrictive abortion laws, in recent years several countries have liberalized abortion access. Newly reformed countries would benefit from having better information on likely barriers and facilitators of implementation, yet studies documenting the implementation of law change soon after it has occurred are rare. Colombia and Mexico are two countries that have recently decriminalized abortion at the national level. While a few studies looked at service implementation following Mexico City’s decriminalization in 2007 [32,33], no studies have yet been conducted to systematically assess progress in implementation after Mexico’s or Colombia’s recent national law reform [34]. This study in Argentina was undertaken because it was considered useful to measure change after an early phase of implementation, to assess gaps and challenges two years after the 2021 abortion law reform. While Colombia and Mexico have different political and legal contexts and systems for administering health service delivery than Argentina, the present study may help to stimulate interest in conducting similar studies in Colombia and Mexico.

This study examined the first two years of implementation of the 2021 abortion law reform in Argentinian public health facilities in three provinces, offering valuable insights to inform public policies aimed at improving access to legal abortion services. The promising progress documented in this study is the result of a committed collaborative effort between national and provincial governments, service providers and civil society organizations following a long history of social mobilization around abortion law reform [16,35–38]. In particular, it was facilitated by the large-scale societal support fostered by the Green Wave movement that later spread across the region [39], resulting in abortion liberalization in two more countries since 2021: Mexico and Colombia [40]. However, the road to abortion reform in Argentina has been a long and bumpy one, with conservative opposition exerting strong pressure throughout the last four decades, often successfully slowing or halting progress [41]. Given Argentina’s position as a leader in mobilization of broad-based support for abortion law reform in the face of persistent and well-organized opposition [42], newly reformed Latin American countries and others considering abortion liberalization are likely to turn to Argentina as a model, taking stock on successful implementation strategies based on their experience. When it comes to local-level implementation, Argentina’s federal political system presents opportunities and barriers that may be relevant to other federal countries in the region, such as Brazil and Mexico [43]. It is therefore crucial to carefully document successes and challenges so others can learn from them. Below, we highlight key findings and their implications around four themes.

### Integration of abortion services into the public healthcare system

Less than two years after law reform, a large number of public facilities were successfully offering abortion services. According to the National Department of Sexual and Reproductive Health (DNSSR), the number of abortion-providing facilities increased from 907 in 2020 to over 1,900 in 2023 [24]. The speed of implementation is all the more notable given the context of the Covid-19 pandemic and the numerous challenges it engendered [18]. According to one study, this rapid expansion of service provision was facilitated by providers’ experience performing abortions under the specific indications of the previous law, as well as strong political will and commitment from health authorities and growing destigmatization of abortion [16]. The role of social and feminist organizations and professional networks in this process should not be underestimated: in addition to campaigning, they were active in disseminating information on safe and friendly abortion providing facilities throughout the country. Some of these organizations offered medical training, counseling and accompaniment of women throughout the abortion process [44,45]. Following abortion legalization, they continue to be key contributors through monitoring law implementation, organizing information campaigns, and running hotlines. With the recent change of government, which has implied drastic cuts to the health system and interruptions in the supply of medication, civil, feminist and professional organizations are once again assuming a critical role in guaranteeing and monitoring access to safe abortion. By encouraging the publication of statistics on abortions throughout the country, networks such as the Red de Socorristas are helping to give the practice more visibility; such strategies are key to breaking the silence on abortion that kept the procedure clandestine for so long, despite its legality [46].

We found that statistical records have been greatly improved, with the majority of surveyed facilities collecting data on abortion services, although the SILVE data system is still not widely used. In addition to giving more visibility to abortion, robust data systems are key to ensuring implementation barriers can be swiftly addressed. Ongoing monitoring initiatives based on official statistics, such as *proyecto mirar*, are crucial [24,47,48]; in addition, regularly conducted facility-based surveys such as ours can assess service provision quality on the ground. In Ireland, the 2018 abortion law reform included reporting requirements to review implementation after three years [49]. Introducing similar requirements for Argentina may help accelerate progress in service provision and also institutionalize reporting, which is particularly pressing in the context of a new conservative government with little interest in guaranteeing provision of abortion services [50]. Rendering the practice invisible through lack of financial support, training, and statistical reporting has been a strategy used by conservative governments to increase stigma and “silence abortion” [46].

Full roll-out of abortion services is likely to take some time: many facilities that would be expected to provide abortion did not do so, and relatively few of the non-providing facilities surveyed planned to offer abortion in the future. This situation is likely to get worse as the new government deprioritizes sexual and reproductive healthcare in funding allocation for health services [51]. Abortion-providing facilities were already particularly scarce in the poorer and more remote province of Chaco. Too few providers in rural areas was also reported in recent reviews of implementation [16,37,47], and was similarly highlighted as a barrier to access in Ireland following the 2019 law reform [52]. While this is partly due to a lack of trained providers, particularly for second trimester abortions [16], we found that a major reason for the patchy coverage is the organization of referral systems that concentrate abortion provision in a few hospitals across each province, as a means of more efficiently distributing resources and supplies to facilities. While this may be an efficient way to organize service provision, key informants noted challenges with the system, and it is unclear whether it effectively meets demand in areas far from the closest referral hospital. In Ireland, the START group connects smaller providers across the country, ensuring a support network in more sparsely populated rural areas [52]. In Argentina, the decision to concentrate abortion care in more remote areas in hospitals rather than distributing it across smaller providers, may jeopardize access for those far from these hospitals. Relatedly, when the national government provides resources for abortion services, provinces must allocate these resources for that end. However, if the national government reduces support for abortion services, poorer provinces such as Chaco with historically conservative values and strong resistance to abortion may be more prone to making cuts to human and financial resources for abortion care. This may further reduce the coverage of providers in the referral system in the areas where they are already most scarce.

### Accessibility of abortion services

Before law reform, individuals seeking an abortion regularly had to overcome several obstacles to accessing abortion, including the requirement for police reports in rape cases and unnecessary delays in procedures [53]. These barriers were often the result of informal rules which, although not (or no longer) legally required, were enforced through public pressure, effectively restricting access to abortion without having to pass any law to this effect [41]. We found that less than two years into law reform, users could access abortion services privately, confidentially, and in a timely manner. This “proceduralization of abortion regulation” [41] afforded by the new law appears to be at least partly succeeding in dismantling the informal rules that governed abortion access until then.

A key mechanism for improving service access is making potential service users aware of the service and their rights. KIS respondents highlighted the lack of mass communication about the new law, while facilities reported that many patients lack information on their rights and types of procedures available. *Proyecto mirar* similarly found a lack of public information disseminated by the government [16]. While information is made available via targeted channels, including hotlines [15,54] and civil society initiatives [16], there is little communication to the general public. Dissemination of accurate information about the law is particularly important in preventing informal rules from taking hold as they have in the past. Such informal rules were often facilitated by a lack of clarity around certain aspects of the law, or the general population’s lack of knowledge of it, which anti-abortion groups would use to their advantage to push for the most conservative interpretations of the law [41]. Public communication campaigns are therefore critical in ensuring correct implementation.

### Availability of supplies, equipment and training

Progress is also seen in the provision of misoprostol by trained professionals in all abortion-providing facilities, with minimal stock-out issues. The DNSSR increased its distribution of misoprostol treatments from 18,500 in 2020 to around 70,000 per year following law reform [24]. With mifepristone’s official approval in March 2023 [55], use of the combined misoprostol-mifepristone treatment is expected to increase; however, training is currently lacking, particularly in health centers. Only half of health centers had personnel trained in MVA, despite many expressing an interest in providing this service, and many health centers do not have MVA kits, since MVA procedures are not expected to be performed by this level of facility. Integrating a wide range of techniques into training protocols will ensure patients have a choice of methods, as recommended by the WHO abortion care guideline [13]. Finally, while all facilities offered post-abortion contraceptive counseling, including a wide range of free methods, contraceptive uptake still needs to be improved, especially in lower-level facilities.

### Availability of personnel

Over a third of province-level hospitals and health centers reported that they did not have sufficient staff to meet demand. Lack of trained staff was the second most cited reason for not offering abortion at all, and was identified by KIS respondents as the greatest obstacle to implementation. While lack of trained staff is partly due to inadequate training of *available* staff, it is also due to insufficient numbers of doctors, compounded by the high number of conscientious objectors, particularly in hospitals. A recent study also found high levels of conscientious objection (CO), particularly among hospital-based providers and those working in more remote provinces [56]. Although CO is permitted only for doctors, other staff frequently invoke it. Such non-compliance is likely symptomatic of a form of institutional weakness characteristic of Latin American countries like Argentina that have suffered decades of political and economic instability. In their analysis of institutional weakness, Brinks et al. identify non-compliance with the law as one of three types of weakness [57]. The prevalence (at least until recently) of informal rules around abortion may be another symptom of this type of institutional weakness, where state officials do not or cannot adequately enforce the law. While the new law presents an opportunity for establishing processes that would increase compliance with abortion regulations, strengthening institutions often takes time, and the arrival of the new conservative government in 2024 may instead risk further destabilizing the institutions in question. Strategies to address non-compliance around CO should consider this wider institutional context.

While CO did not usually lead to denial of procedures by abortion-providing facilities, it reduces the number of available staff and increases the workload of providing doctors. CO appears to be even more problematic in non-providing facilities, and was frequently cited as a reason for not providing abortions in La Rioja and RPBA. Similar consequences of CO on workloads were also noted in Ireland after the 2019 law reform, where providers complained that the high levels of CO, particularly in hospitals (as in Argentina), resulted in an unmanageable workload for those who did agree to provide, and that while there appeared to be extensive support for objectors, there was not enough institutional support for abortion providers [52]. In Argentina, a 2020 study predating law reform found that CO was often used by hospital authorities as an ideological tool to impose conservative political agendas, while individual staff tended to use CO not for moral reasons, but as a protective mechanism for fear of stigma or legal repercussions should they perform abortions [58]. Indeed, abortion providers in Argentina have historically been seen as less legitimate than conscientious objectors, and subjected to harassment and discrimination [5]. Yet, there is also evidence that abortion law reform has helped to de-stigmatize the procedure, in particular among medical providers [16]. Already before the recent law reform, interviews with providers in Buenos Aires between 2014 and 2017 (following a 2012 Supreme Court ruling that clarified the legal grounds for abortion) found that providers were beginning to talk about abortion more openly and with more pride in their work, as the abortion debate reached greater public prominence and reduced the stigma around the practice [12]. Being part of a network, such as the “Network of health professionals for the right to decide”, also helped providers to modify their perceptions of stigma [59]. However, stigma tends to persist even after the procedure has been legalized. A study of abortion patients and providers in Uruguay two years post-decriminalization found that stigma continued to restrict access to abortion despite its legality, for example through mandatory waiting times, lack of choice of method, or disapproval from providers [60].

Understanding providers’ motivations for not wanting to provide abortions may help point to other possible solutions to staff shortage. For example, another reason for CO cited in the 2020 study was avoidance of heavy workloads; if this is still the case, offering better incentives to prospective doctors and other staff to perform abortions (as proposed by KIS respondents) may increase the pool of providers. In parallel, authorizing and training midwives to perform first trimester abortions would help fill the capacity gap, and a bill to this effect is currently making its way through Congress [16]. Finally, the recent review by *proyecto mirar* found that the general lack of oversight of CO enables professionals to claim it without following official regulations, such as prompt referral to consenting providers [16]. In another recent study, providers – and particularly conscientious objectors – erroneously believed more requirements needed to be fulfilled for on-request abortions, such as judicial authorization for minors or HIV tests, which can unnecessarily delay or obstruct patients’ timely access to abortion [56]. Extending training to non-medical staff on abortion law and guidelines regarding CO could reduce the number of objectors [19,58]. Although at the time of the study, there were no plans to strictly enforce CO regulations, there was no consensus among respondents about whether enforcement would have positive results. Instead, based on opinions from some respondents, it would appear that a more nuanced regulation on CO could be beneficial, for example permitting objection to later gestation abortions only, as opposed to the current situation where CO is a blanket objection to all abortions.

### Study limitations

This study is not representative at the national level. The research was carried out in three provinces selected with an eye to diversity, and as such provides a snapshot of different situations in the country. However, the small number of abortion-providing facilities in Chaco limited our ability to generate estimates for this province. Second, since the key informant survey data are based on respondents’ opinions, this provides indirect and subjective information on the state of implementation. However, key informants’ lengthy experience and expertise on this topic make their opinions valuable for understanding challenges to implementation. Third, given that this study was focused on the supply side of abortion provision, we did not examine the perspectives and experiences of service users themselves: all information presented on patients is based on health providers’ perceptions. Further research on patients’ experiences accessing abortion, and more generally on people’s needs in relation to abortion services, could help identify demand-side barriers to obtaining abortions, and strategies to address them. A few studies have described people’s experiences and preferences seeking abortions prior to 2021 [6,61,62], but evidence is lacking on service users’ experiences and needs following law reform.

## Conclusion

The first two years following abortion law reform in Argentina have seen significant progress in increasing access to abortion services, especially given the Covid-19 pandemic and limited resources. The biggest remaining challenge is around availability of trained personnel, including the regulation of conscientious objection. We propose the following recommendations to further improve abortion provision.

1) Increase the number of providers to meet demand: Encourage more doctors to provide abortion by offering incentives such as free training and improved working conditions, and counteracting stigma; authorize and train midlevel staff such as midwives and nurses to provide early abortions without needing a doctor to sign off; organize compulsory training on the limits of CO for all staff (including non-medical) involved in abortion provision; and more strictly enforce CO regulations.
2) Improve the quality of abortion services: Increase training on safe abortion methods such as the misoprostol-mifepristone combination and MVA; integrate contraceptive counseling early in the abortion process rather than waiting till the post-abortion follow-up appointment; and monitor the effectiveness of referral circuits in meeting demand in more remote provinces.
3) Carry out public information campaigns covering the right to abortion, how to access the service and available methods.

Investing in these strategies, along with more research on service users’ needs and on how to address conscientious objection, and ongoing monitoring of implementation progress in facilities, can help improve abortion provision and inform guideline updates, to ensure that comprehensive services are available to all those who need them. This is particularly relevant given that the new government that took office in December 2023 has openly expressed opposition to abortion [63] and has already begun to implement substantial budget cuts in public programs, including those that support reproductive healthcare [64,65]. Argentina’s experience implementing abortion law reform offers useful guidance to stakeholders in other countries that have or are seeking to liberalize their abortion laws.

## Supporting information

S1 Data Health facilities Survey dataset. (XLSX)

S2 Data Key Informant Survey dataset. (XLS)

S1 Text Inclusivity in global research questionnaire. (DOCX)
